# A Computational Model for Aperture Control in Reach-to-Grasp Movement Based on Predictive Variability

**DOI:** 10.3389/fncom.2015.00143

**Published:** 2015-12-10

**Authors:** Naohiro Takemura, Takao Fukui, Toshio Inui

**Affiliations:** Department of Intelligence Science and Technology, Graduate School of Informatics, Kyoto University, Yoshida-honmachiKyoto, Japan

**Keywords:** motor control, reach-to-grasp movement, online vision, computational model, Kalman filter

## Abstract

In human reach-to-grasp movement, visual occlusion of a target object leads to a larger peak grip aperture compared to conditions where online vision is available. However, no previous computational and neural network models for reach-to-grasp movement explain the mechanism of this effect. We simulated the effect of online vision on the reach-to-grasp movement by proposing a computational control model based on the hypothesis that the grip aperture is controlled to compensate for both motor variability and sensory uncertainty. In this model, the aperture is formed to achieve a target aperture size that is sufficiently large to accommodate the actual target; it also includes a margin to ensure proper grasping despite sensory and motor variability. To this end, the model considers: (i) the variability of the grip aperture, which is predicted by the Kalman filter, and (ii) the uncertainty of the object size, which is affected by visual noise. Using this model, we simulated experiments in which the effect of the duration of visual occlusion was investigated. The simulation replicated the experimental result wherein the peak grip aperture increased when the target object was occluded, especially in the early phase of the movement. Both predicted motor variability and sensory uncertainty play important roles in the online visuomotor process responsible for grip aperture control.

## Introduction

One of the noted behavioral features of primates is their ability to use their hands to interact with objects in various situations. Motor outputs that control the hands are generated based on environmental information collected through sensory inputs. The central nervous system (CNS) computes a suitable transformation of these sensory inputs to motor outputs that allows motor performance required in daily life. One of these performances is the reach-to-grasp movement, in which the primate extends the arm toward an object placed in front of it, and then grasps the object with its fingers.

Jeannerod (1981, 1984) has investigated behavioral properties associated with these prehension movements. In his experiments, human participants were instructed to perform natural reach-to-grasp movements toward visually presented target objects of several sizes placed at several distances. He found some basic features: the velocity profile of the hand exhibits one peak in the first half of the movement duration, while the grip aperture demonstrates one peak in the second half. The peak grip aperture (PGA) is scaled to the size of the target object (e.g., Marteniuk et al., 1990). Appropriate transformation from the visual perception of the target object into the generation of the PGA is required for successful prehension movement.

How the CNS computes this transformation from sensory input of the visual object size to motor output in the form of a PGA is not yet clear. Previous studies have reported that the PGA is larger when vision is not available during the movement (e.g., Wing et al., 1986; Jakobson and Goodale, 1991; Fukui and Inui, 2006) or when the movement speed is particularly rapid (Wing et al., 1986). One interpretation of these observations is that the grip aperture is controlled to prevent inappropriate collisions of the fingers with the target and that the PGA becomes larger when visual uncertainty and/or motor variability are increased because of visual occlusion and/or faster movement (Wing et al., 1986). Actually, if the target object is presented in an eccentric view and its actual position is uncertain, the PGA will increase linearly with the eccentricity of the view (Schlicht and Schrater, 2007), which indicates that visual uncertainty is influential in the mechanism for grip aperture control. Elucidating the underlying mechanism for this grip aperture control would be possible with the use of appropriate modeling studies. Although previous models have described the reach-to-grasp movement (Hoff and Arbib, 1993; Haggard and Wing, 1995; Zaal et al., 1998; Smeets and Brenner, 1999; Ulloa and Bullock, 2003; Simmons and Demiris, 2006), no model has yet satisfactorily explained the effect of visual uncertainty on the generation of the PGA.

Traditionally, one of the major problems in modeling motor control is determining one trajectory of movement in the presence of motor redundancy. A prevailing idea is that the CNS solves this problem by minimizing costs to generate these movement trajectories, and several costs have been proposed (e.g., Flash and Hogan, 1985; Uno et al., 1989; Harris and Wolpert, 1998; Todorov and Jordan, 2002). One index of the cost minimizations that successfully explains human reaching trajectory is movement smoothness (Flash and Hogan, 1985; Uno et al., 1989). However, why the CNS adopts such costs is not clear. Harris and Wolpert (1998) focused on the importance of the cost of the final variance, because it is clearly related to the achievement of the task. If the final body state is less variable, the movement is more likely to be successful. Motor control also needs to optimize the possibility of task achievement in the presence of motor variability (Miyamoto et al., 2004; Todorov, 2004; Trommershäuser et al., 2005). Thus, recent computational models of motor control have emphasized the importance of both sensory and motor variability (e.g., van Beers et al., 2002, 2004; Guigon et al., 2008).

In a reach-to-grasp movement, the compensation for motor variability would result in adjustment of the PGA. A minimal variance model proposed by Simmons and Demiris (2006) indeed explains the emergence of PGA in reach-to-grasp movement. Specifically, their model reported that a higher movement speed in a reach-to-grasp movement creates a larger motor variability, and the grip aperture increases in a compensatory manner to prevent undesirable collisions of the hand with the target. However, since visual occlusion of the target and/or of the hand would affect sensory uncertainty, not motor variability, the manner how sensory feedback contributes to variability-based motor control should be revealed.

To clarify this manner, Todorov and Jordan (2002) proposed a computational model for motor control based on stochastic control theory. In their model, called optimal feedback control, a copy of the motor command and the noisy sensory feedback are used for internal estimation of the current state of the body. The optimal motor command that minimizes the task cost is then computed from this state estimation. The body state is estimated based on both motor and sensory variability, and the motor command is generated by taking into account the uncertainty of the state estimation. The concept underlying this computational model could explain the effect of online vision during reach-to-grasp movement, as it gives sensory feedback the role of compensating for motor variability and improving the estimation of the current body state. Specifically, when visual feedback is absent, the motor variability would directly cause uncertainty in the estimated body state due to the loss of the compensation by sensory feedback. Our hypothesis is that a larger PGA would appear as a result of compensating for the increased uncertainty of state estimation.

In the present paper, we propose a stochastic control model for grasping that can simulate the effects of online vision. Although, Todorov and Jordan (2002) verified their own model by simulating arm reaching (pointing) movement, application of their model directly to reach-to-grasp movement is difficult because the movement is too complicated to be described by the task cost that is available in their model. Instead, we avoid optimal control and consider “compensation for sensory and motor variability” for calculating motor command, thereby preserving the concept of stochastic control.

An argument could be made that the effect of visual occlusion during grasping might be explained without invoking an online control mechanism that includes motor and sensory variability; i.e., some strategic effect against removing vision (e.g., the visual feedback schedule) might be causing a larger PGA (Jakobson and Goodale, 1991). However, Fukui and Inui (2006) investigated the temporal and spatial effects of visual occlusion on the grip configuration when several occlusion conditions were randomly presented (see also Whitwell et al., 2008) and found that removing the target vision in the early phase of the movement enlarged the PGA, which indicated that the PGA was modified by the availability of online vision, not only by the motor strategy. Here, we verify that the effect of visual occlusion on the PGA is due to an online control mechanism that includes motor and sensory variability by simulating the experiments of Fukui and Inui (2006) using the following model.

## Materials and methods

### Overview of the aperture control model

Reach-to-grasp movement is traditionally thought to have two control components: a transportation (reaching) component and an aperture (manipulation) component (Jeannerod, 1981). Although the kinematics of trajectories of each finger are different, which may result from detailed control of each finger (Smeets and Brenner, 1999), the grip aperture size that represents the spatial relationship of these fingers shows significant correlation with the object size (Marteniuk et al., 1990).

Schlicht and Schrater (2007) suggested, based on the principal components analysis, that only one principal component, which can be represented by the aperture size, would explain the difference in the trajectory of the fingers during reach-to-grasp movements where the target is placed in different visual eccentricities. Therefore, we assume that modeling grip aperture between thumb and index finger, and not the kinematics of each finger, is sufficient for revealing the grip control mechanism influenced by online vision (see also Vilaplana and Coronado, 2006).

**Figure 1:**
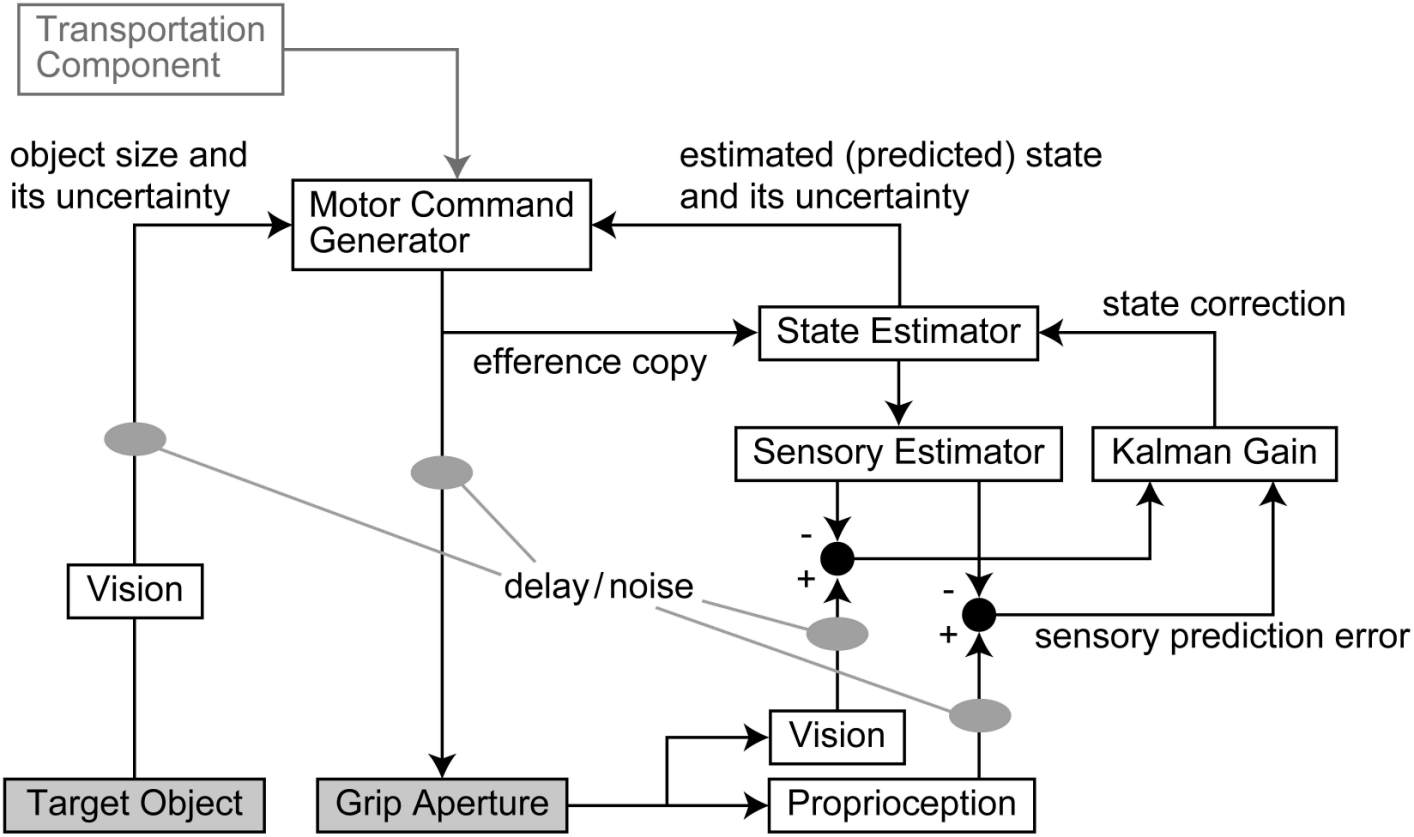
**Outline of the model**. The output motor command that controls grip aperture is transmitted with delay and noise. At the same time, an efference copy of the motor command is transmitted to the State Estimator, and the body state (grip aperture) is estimated (or predicted) by the forward model. The sensory prediction is computed from the estimated aperture, and is compared with the sensory feedback (vision and proprioception) of the actual grip aperture. The Kalman filter corrects the estimated body state based on the sensory prediction error. The next motor command is generated based on the estimated (predicted) body state and the variability (variance) of estimation. The size and variability (variance) of the target object that are observed by vision with noise and delay are then used to calculate the motor command. Although the transport component was assumed to contribute to the aperture control, the result of parameter fitting showed that it was not effective.

An outline of the model is shown in Figure 1. The output motor command that controls grip aperture is transmitted to the end effector (hand) with delay and noise, and this changes the grip aperture. At the same time, the efference copy of the motor command is transmitted to the State Estimator, with no delay, and the body state (grip aperture) is estimated (or predicted) by the forward model. The estimated aperture is compared with the sensory feedback derived from vision and proprioception of the actual grip aperture, and the estimation error is then used to correct the body state estimation. The next motor command is generated based on the estimated (predicted) body state and the uncertainty of the estimation. The size and uncertainty of the target object that is observed by vision are then used to calculate the motor command. Further details are described below.

### Aperture size to compensate for sensorimotor variability

**Figure 2:**
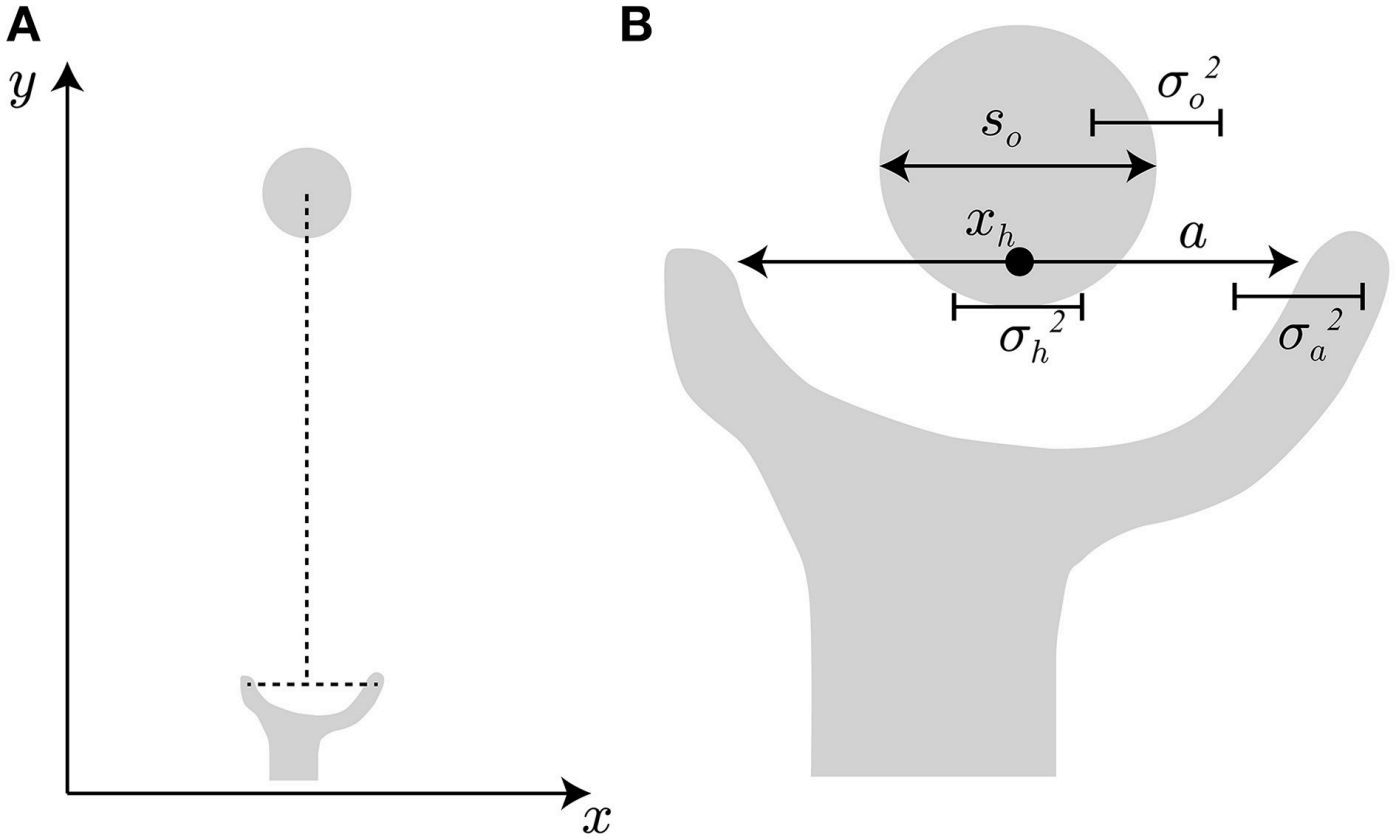
**Hand and target object. (A)** Hand transportation follows the *y*-axis, and grip opening follows the *x*-axis. **(B)** For successful grasping, the grip aperture *a* must be sufficiently larger than object size *s*_*o*_, considering the variability of grip aperture $\sigma_{\text{a}}^{\text{2}}$, hand position $\sigma_{\text{h}}^{\text{2}}$, and target object $\sigma_{\text{o}}^{\text{2}}$.

In the model setting, the hand travels from the start position to the object position along the y-axis, as indicated in Figure 2A. This hand transportation is not modeled here. The transportation component (the profile of hand position along the y-axis) is realized by the data from the experiment by Fukui and Inui (2006). The finger aperture opens along the x-axis because the target object is a simple cylinder and the grip orientation has little effect on the aperture size. When the transportation component properly controls the hand position so that the center point between the thumb and finger matches the center of the target object, the problem becomes how the aperture size is controlled as the hand approaches the object.

To avoid an undesirable collision during a reach-to-grasp movement, the grip aperture has to include a margin of safety that accounts for uncertainty of the hand and the object. For example, consider the situation shown in Figure 2B to represent the beginning of the finger closure phase of the movement. If the position on the x-axis and the diameter of the object are denoted by *x*_*o*_, *s*_*o*_ respectively, the position of the right edge of the object is *x*_*o*_ + *s*_*o*_∕2. Similarly, the position of the finger at the right side of the object (i.e., the index finger) is *x*_*h*_ + *a*∕2, if the hand position (the center point between the thumb and the finger) and the aperture size are denoted by *x*_*h*_ and *a*, respectively. To avoid collision of the finger with the object, the finger position has to be to the right of the object edge, i.e., *x*_*o*_ + *s*_*o*_∕2 < *x*_*h*_ + *a*∕2. We assume that *x*_*o*_ − *x*_*h*_ = 0 because the hand position should be controlled to match the object position by the transportation component. However, the motor variability and sensory uncertainty of hand position relative to the object position still exists, and here, the standard deviation (SD) of the variability (uncertainty) is denoted by σ_*h*_. The variability (uncertainty) of the aperture size and the object size (the SDs of them are denoted by σ_*a*_ and σ_*o*_) also take effect. The situation before successful grasping has to satisfy *a* − *s*_*o*_ >0 under the condition where the variability of *a* − *s*_*o*_ is characterized by the variance $\sigma^{2}=\sigma_{a}^{2}+\sigma_{h}^{2}+\sigma_{o}^{2}$, if Gaussian distribution and the independence of these variabilities is assumed. The same situation takes place at the left side of the object. Here, the probability Φ (*a, s*_*o*_, σ) that the target object is inside the grip aperture is formulated by:

(1) $$\begin{array}{l} \Phi \left(a,s_{o},\sigma \right)=\frac{1}{\sqrt{2\pi \sigma^{2}}}\int_{0}^{\infty }\text{exp}\frac{{-\left(a^{\prime }-\left(a-s_{o}\right)\right)}^{2}}{2\sigma^{2}}da^{\prime } \end{array}$$

This is the cumulative distribution function that describes the probability that the grip aperture *a* is larger than target object *s*_*o*_ in the presence of Gaussian noise.

In order to execute a grasping movement when uncertainty is involved, the CNS has to take the risk of collision into account. If we consider a particular probability ϕ, which the CNS adopts as a success ratio, the grip aperture to achieve is then calculated by $a^{*}=\Phi^{-1}\left(\phi ,s_{o},\sigma \right)$. Note that ϕ is the probability that the CNS predicts before executing the movement with the uncertainty and variability (i.e., σ^2^) that the CNS estimates, and that ϕ does not represent the actual success rate of the grasping. Here, we call the aperture a^*^ the *target aperture*. Once the target aperture is determined, the controller generates a motor command that moves the current grip aperture toward the target aperture.

Among infinite possibilities of motor commands that make the current grip aperture approach the target aperture, one motor command should be determined to generate an actual movement. In human arm control, the movement trajectory is determined by minimizing a certain criterion (Flash and Hogan, 1985; Uno et al., 1989; Harris and Wolpert, 1998). Smoothness of movement is an important factor in determining the trajectory; the trajectory of point-to-point movement starting and ending with zero velocity and zero acceleration is well explained by the minimum jerk model (Flash and Hogan, 1985). However, the start point of the trajectory formulation required in the present model is not the movement start point but the *current* point during movement, and the initial velocity and acceleration of the trajectory are not zero. Minimum acceleration, which is another criterion for kinematic smoothness of movement, also well explains the reaching trajectory if boundary conditions (i.e., the initial and final velocity and acceleration) are specified (Ben-Itzhak and Karniel, 2008). Here, we adopted the minimum acceleration criterion for generating a motor command (Note that the model only generates the *next* motor command from the *current* grip aperture, not the whole trajectory).

To achieve successful grasping, the thumb and fingers have to attain a suitable contact with the surface of the object. At the same time, they have to avoid undesirable collision with the object. The relative timing of these requirements is different: first, for avoiding collision, and then, for contacting the object. Winges et al. (2003) investigated finger formation for different shaped objects during reach-to-grasp movement under several visual conditions, and they concluded that visual occlusion prolonged the final low-speed phase of reaching when the precise finger formation occurred. Our model is focused on the mechanism for generating peak grip aperture, not for contacting the object; therefore, we did not implement the mechanism for closing fingers upon suitably touching the object surface. Details are mentioned in the section that covers the simulation (Section Simulation).

### Formulation of aperture size control

The movement of grip aperture is modeled by a state-space representation:

(2) $$\begin{array}{l} {\text{x}}_{t+\Delta t}=F{\text{x}}_{t}+Du_{t}+Gw_{t}\\ F=\left[\begin{array}{cc} 1 & \Delta t\\ 0 & 1 \end{array}\right]\;D=\left[\begin{array}{c} 0\\ \Delta t \end{array}\right]\;G=\left[\begin{array}{c} 0\\ 1 \end{array}\right] \end{array}$$

Here, ${\text{x}}_{t}={\left[a_{t},{ȧ}_{t}\right]}^{T}$, where *a*_*t*_ and ȧ_*t*_ is grip aperture and its velocity at time *t*, respectively, and [□]^*T*^ denotes transposition of a vector or matrix. Motor command *u*_*t*_ is acceleration of grip aperture and *w*_*t*_ is the motor noise generated from *N*(0, *Q*_*t*_), while Δ*t* is a discrete time step in simulation. The controller generates motor commands to achieve the target aperture a^*^ at the movement end time *T*. As a requirement in the experiment, the movement duration is predefined to be 1 second (Fukui and Inui, 2006). In order to simulate a smooth movement that achieves target aperture a^*^ and target aperture velocity ȧ^*^ = 0 at the movement end, we adopted the minimum acceleration criterion. The velocity at time τ (*t* ≤ τ ≤ *T*) in the minimum acceleration profile is described by:

(3) $$\begin{array}{l} {ȧ}_{\tau }={ȧ}_{t}+b_{1}\left(\tau -t\right)+b_{2}{\left(\tau -t\right)}^{2} \end{array}$$

(4) $$\begin{array}{l} b_{1}=\frac{6\left(a^{*}-a_{t}\right)}{{\left(T-t\right)}^{2}}-\frac{4{ȧ}_{t}}{T-t} \end{array}$$

(5) $$\begin{array}{l} b_{2}=-\frac{6\left(a^{*}-a_{t}\right)}{{\left(T-t\right)}^{3}}-\frac{3{ȧ}_{t}}{{\left(T-t\right)}^{2}} \end{array}$$

(See Appendix A). What the controller determines here is not the whole trajectory, but the instant acceleration at time *t*. Based on Equation (3), the aperture velocity at time *t* + Δ*t* is:

(6) $$\begin{array}{l} {ȧ}_{t+\Delta t}={ȧ}_{t}+b_{1}\Delta t+b_{2}\Delta t^{2} \end{array}$$

Since ȧ_*t* + Δ*t*_ = ȧ_*t*_ + *u*_*t*_Δ*t* comes from Equation (2), the motor command at time *t* in the minimum acceleration criterion is:

(7) $$\begin{array}{l} u_{t}=b_{1}+b_{2}\Delta t \end{array}$$

Although, the motor command is calculated from the current and target aperture, the CNS does not know the true state of the current body. The information about body state has to be obtained by the sensory system. Here, grip aperture is observed by vision and proprioception:

(8) $$\begin{array}{l} {\text{y}}_{t}=H{\text{x}}_{t}+{\text{v}}_{t}\\ H=\left[\begin{array}{cc} 1 & 0\\ 1 & 0 \end{array}\right] \end{array}$$

For simplicity, we assumed that only the grip aperture (not the aperture velocity) is used as input information. Here, the first element of ***y***_*t*_ is for vision and the second one is for proprioception, where ***v***_*t*_ is observation noise generated from *N*(0, *R*_*t*_). The Kalman filter (Appendix B) estimates the body state from sensory observation. If the estimated body state is ${\hat{\text{x}}}_{t}={\left[{â}_{t},{\hat{ȧ}}_{t}\right]}^{T}$ then *a*_*t*_ and ȧ_*t*_ in Equations (4) and (5) are replaced by â_*t*_ and ${\hat{ȧ}}_{t}$, respectively:

(9) $$\begin{array}{l} {\hat{b}}_{1}=\frac{6\left(a^{*}-{â}_{t}\right)}{{\left(T-t\right)}^{2}}-\frac{4{\hat{ȧ}}_{t}}{T-t} \end{array}$$

(10) $$\begin{array}{l} {\hat{b}}_{2}=-\frac{6\left(a^{*}-{â}_{t}\right)}{{\left(T-t\right)}^{3}}-\frac{3{\hat{ȧ}}_{t}}{{\left(T-t\right)}^{2}} \end{array}$$

These equations generate the motor command based on the estimated body state.

The actual brain-body system undergoes transmission delays in the motor command and the sensory feedback pathway. When motor delay and sensory delay are *d*_*m*_ and *d*_*s*_, respectively, Equations (2) and (8) are modified to:

(11) $$\begin{array}{l} {\text{x}}_{t+\Delta t}=F{\text{x}}_{t}+Du_{t-d_{m}}+Gw_{t} \end{array}$$

(12) $$\begin{array}{l} {\text{y}}_{\text{t}}=H{\text{x}}_{t-d_{s}}+{\text{v}}_{t} \end{array}$$

The motor command generated at time *t* has to be based on the body state (estimation) at time *t* + *d*_*m*_. Here, Equations (9) and (10) are modified to:

(13) $$\begin{array}{l} {\hat{b}}_{1}=\frac{6\left(a^{*}-{â}_{t+d_{m}}\right)}{{\left(T-\left(t+d_{m}\right)\right)}^{2}}-\frac{4{\hat{ȧ}}_{t+d_{m}}}{T-\left(t+d_{m}\right)} \end{array}$$

(14) $$\begin{array}{l} {\hat{b}}_{2}=-\frac{6\left(a^{*}-{â}_{t+d_{m}}\right)}{{\left(T-\left(t+d_{m}\right)\right)}^{3}}-\frac{3{\hat{ȧ}}_{t+d_{m}}}{{\left(T-\left(t+d_{m}\right)\right)}^{2}} \end{array}$$

These equations imply that the “current” body representation in the brain precedes the actual body state. This function is necessary for predictive remapping (Colby and Duhamel, 1996; Melcher, 2007), where neural representations of planned saccade movements are modified to compensate for future changes of eye position. Similarly, the grip aperture also has to be predicted before the motor command reaches the effector during reach-to-grasp control.

### Prediction of uncertainty

As mentioned in Section Aperture Size to Compensate for Sensorimotor Variability, the prediction error variance at the end of the movement is necessary for determining the target aperture. The variance of grip aperture $\sigma_{a}^{2}$ is obtained by prediction of the Kalman filter (Kalman, 1960; see Appendix B). The Kalman filter predicts the future body state following the state-space representation, Equation (11), as well as the variance of prediction error. Although, in the Kalman filter, the variance is typically used to determine filter gain for correction of an estimated body state based on sensory feedback, the proposed model also uses the calculated estimation variability for aperture control.

The prediction of the SD of hand position σ_*h*_ is assumed to be proportional to the distance of residual hand transportation. That is:

(15) $$\begin{array}{l} \sigma_{h}=\alpha \left(h^{*}-h_{t}\right), \end{array}$$

where h^*^ is the distance between the initial hand position and the target object, *h*_*t*_ is the distance between the initial hand position and the hand position at time *t*, and α is the proportionality coefficient. This assumption is based on the idea that increases in distance to the target position give rise to more uncertainty in the prediction of final hand position. This agrees with the fact that travel distance and the target width are proportional in Fitts' law (Fitts, 1954). If target width is interpreted as “tolerable size of variability of the final hand position,” then Equation (15) will be derived. As the hand approaches the target position, the prediction of the SD decreases because the residual distance to be traveled decreases.

**Figure 3:**
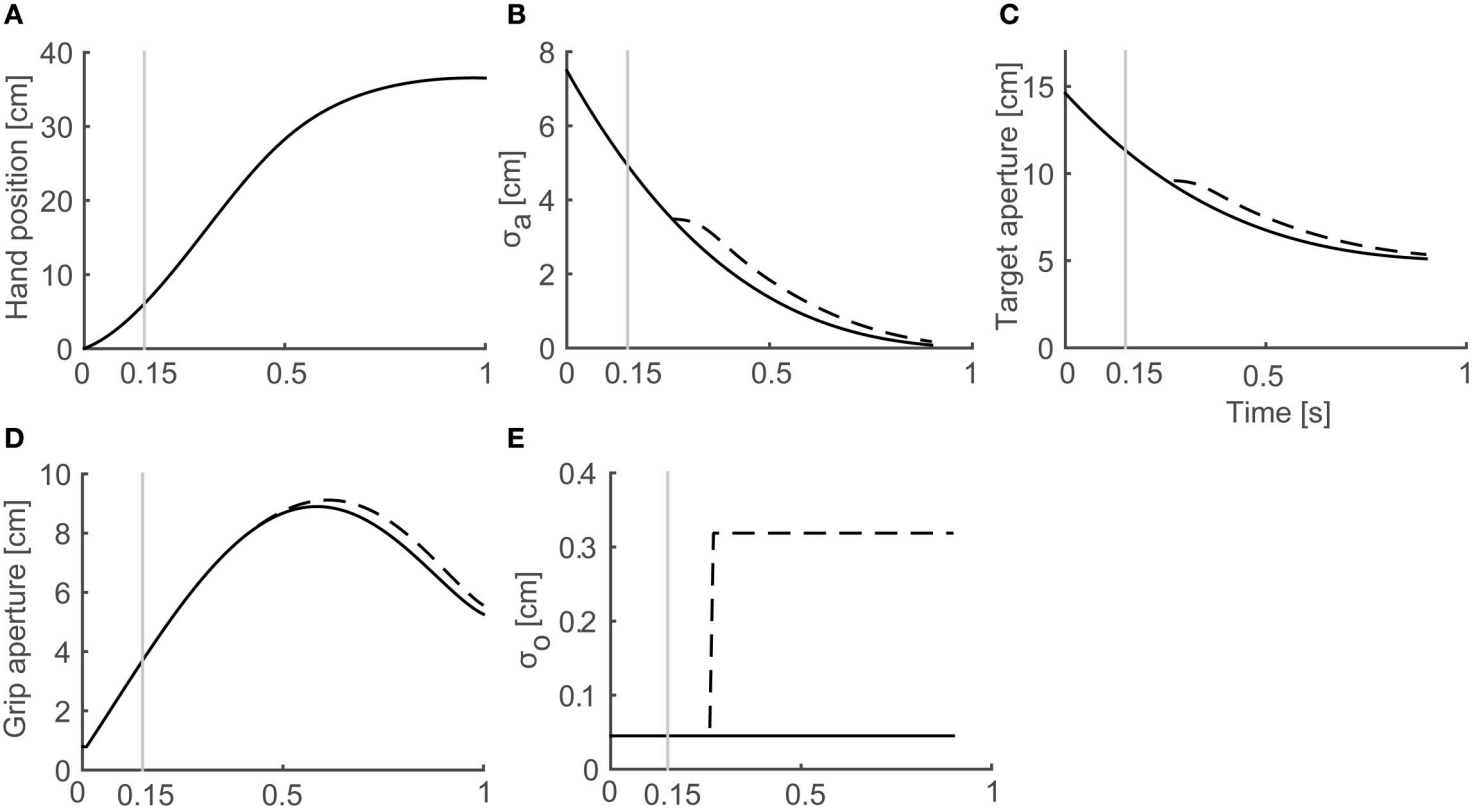
**Profiles of variables in the model**. Variables obtained from a simulation with target object of 4 cm in Full Vision (FV) and 150S (visual occlusion from 150 ms after movement onset) conditions are shown. Black solid and dashed lines denote FV and 150S conditions, respectively. Gray vertical lines denote onset of visual occlusion in 150S condition. **(A)** Hand distance traveled from the start position. **(B)** Predicted SD of grip aperture σ_*a*_. **(C)** Target aperture a^*^. **(D)** Grip aperture. **(E)** SD of object size σ_*o*_.

In a reach-to-grasp movement, the effect of the aperture component on the transportation component is small (Paulignan et al., 1991), especially before the movement end point. Consequently, the use of a fixed transportation component is reasonable for the simulation of the generation of peak grip aperture. Here, we used the wrist position profile (Figure 3A) that is obtained by temporally normalizing and averaging the experimental data of Fukui and Inui (2006).

Finally, because the target object is only observed visually, the variability is determined by visual noise. The variance of uncertainty of the target object size $\sigma_{o}^{2}$ is obtained from the vision element of the covariance matrix of sensory noise *R*_*t*_ (see Section Simulation).

### Simulation

Using the proposed model, we simulated a visual occlusion experiment by Fukui and Inui (2006). In the simulation, movement end time *T* and a discrete time step Δ*t* were set to 1000 and 10 ms, respectively. Both visual and motor delays were set to 100 ms (Huettel et al., 2014). Because the movement onset in the experiment was defined by wrist release from a start position, the grip aperture and the aperture velocity at the movement onset were not zero. Therefore, in the simulation we set the initial grip aperture to be an average grip aperture at the movement onset in all trials recorded in the experiment. Additionally, the initial motor command (acceleration) in the simulation was set to be a value that achieved the average velocity at the movement onset in the experiment. The initial motor command in the simulation was generated taking into account the *d*_*m*_ (motor delay) period preceding movement onset.

For the simulation of visual occlusion during movement, the variance of visual feedback noise was assumed to be larger than usual when vision was unavailable. On the other hand, proprioceptive noise was assumed to be constant. If the variance of visual noise during occlusion (no vision) and during non-occlusion (full vision) are denoted by $\sigma_{NV}^{2}$ and $\sigma_{FV}^{2}$, respectively, and the variance of proprioceptive noise is denoted by $\sigma_{P}^{2}$, the covariance matrix of sensory noise is:

(16) $$\begin{array}{l} R_{t}=\left[\begin{array}{cc} \sigma_{NV}^{2} & 0\\ 0 & \sigma_{P}^{2} \end{array}\right]\;\left(\text{during occlusion}\right)\\ R_{t}=\left[\begin{array}{cc} \sigma_{FV}^{2} & 0\\ 0 & \sigma_{P}^{2} \end{array}\right]\;\left(\text{during non-occlusion}\right) \end{array}$$

No correlation is assumed between visual noise and proprioceptive noise. Viewed from an engineering perspective, visual occlusion should have been simulated by simply setting the Kalman gain to zero. This implementation means that the system estimates the target state only from Equation (2), but we did not adopt this implementation in the current study. Westwood and Goodale (2003) have demonstrated that the grip aperture during visually occluded grasping was affected by a size-contrast illusion, while the grip aperture in normal vision was not. We assume that this may reflect switching of the visual processing pathways from the dorsal “how” stream to the ventral “what” stream (Goodale and Milner, 1992). Therefore, the two kinds of noise assumed in the present model Equation (16) represent these two different types of visual processing.

As described in Equation (2), motor noise is assumed to be a single dimension and affects the system in the same domain as the motor command. This implies that the motor noise originates from motor command. However, the variance of motor noise is assumed to be constant; that is, $Q_{t}=\sigma_{M}^{2}$.

Here, the proposed model has six parameters: the variance of motor noise $\sigma_{M}^{2}$, the variance of visual noise during occlusion and non-occlusion $\sigma_{NV}^{2}$, $\sigma_{FV}^{2}$, the variance of proprioceptive noise $\sigma_{P}^{2}$, the proportionality coefficients α between distance and the variance of hand position, and the probability ϕ that the object has to be inside the aperture. For successful grasping, the probability ϕ should be high. However, if ϕ is too high, the noise variances have to be very small in order to achieve the appropriate grip aperture, and small variances make it difficult to differentiate the visual conditions. Therefore, ϕ was fixed a priori at 0.9 based on the empirical inspection that people could fail prehension performance once out of 10 trials. The other parameters were determined by fitting the model outputs to the experimental data.

A probability ϕ of 0.9 might be rather small for successful grasping. However, the proposed model was not designed to explain precise control of the final phase of the movement, where the successful achievement of grasping is realized by the mechanism for adjusting the aperture size to the object size. Rather, this model aims to demonstrate how the generation of the PGA is influenced by online vision during the movement. We assume that the probability ϕ, which determines the PGA, does not have to be strictly high during the movement. In order to examine the effect of ϕ on the aperture profile, the simulation with various values for ϕ (= [0.8, 0.85, 0.9, 0.95, 0.99]) was also conducted.

### Parameter fitting

Five parameters, σ_*M*_, σ_*P*_, σ_*FV*_, σ_*NV*_ and α, were fixed by fitting the grip aperture profile obtained from the simulation to that in the experiment by Fukui and Inui (2006). In the experiment, a visual occlusion experiment was conducted while the reach-to-grasp movement was in process with two experimental paradigms: a shutting paradigm (SP) and a re-opening paradigm (RP). In the SP, vision was available during target presentation and reaction time phases, and occlusion started immediately after movement onset (NV), or 150 ms (150S), 350 ms (350S), 500 ms (500S), or 700 ms (700S) after movement onset; or vision is never occluded during movement (FV). On the other hand, in RP, occlusion once started at movement onset, and vision was recovered immediately after movement onset (FV), or 150 ms (150R), 350 ms (350R), 500 ms (500R), or 700 ms (700R) after movement onset; or vision was never recovered (NV). Both paradigms had conditions in which vision during the movement was constantly available (FV) or constantly unavailable (NV). Model parameters were fitted against the aperture profile from these constant conditions. For the simulation and comparison of simulated aperture profiles to the ones obtained from the experiment, the movement time of each trial of experimental data was normalized to 1 s, and the grip aperture and wrist position were resampled at 100 Hz by interpolating the experimental data. Note that the participants in the experiment were instructed to keep the movement duration of the trials at approximately 1000 ms. Then, the temporally normalized aperture profiles of all subjects and trials were averaged for each condition.

Fitting was performed by minimizing the squared sum of errors at each time point between the aperture profile of the experiment and the simulations for two constant vision conditions (i.e., FV and NV conditions). Minimization was achieved by the Nelder-Mead simplex method (function *fminsearch* of MATLAB optimization toolbox). The fitting result is shown in Table 1.

**Table 1:** **Results of parameter fitting**.

**σ_***M***_ (cm/ms**^2^**)**	**σ_*P*_ (cm)**	**σ_*FV*_ (cm)**	**σ_*NV*_ (cm)**	**α**
0.3824	0.6043	0.0450	0.3187	0.0000

*Note that the degrees of variability are standard deviation, not variance*.

## Results

**Figure 4:**
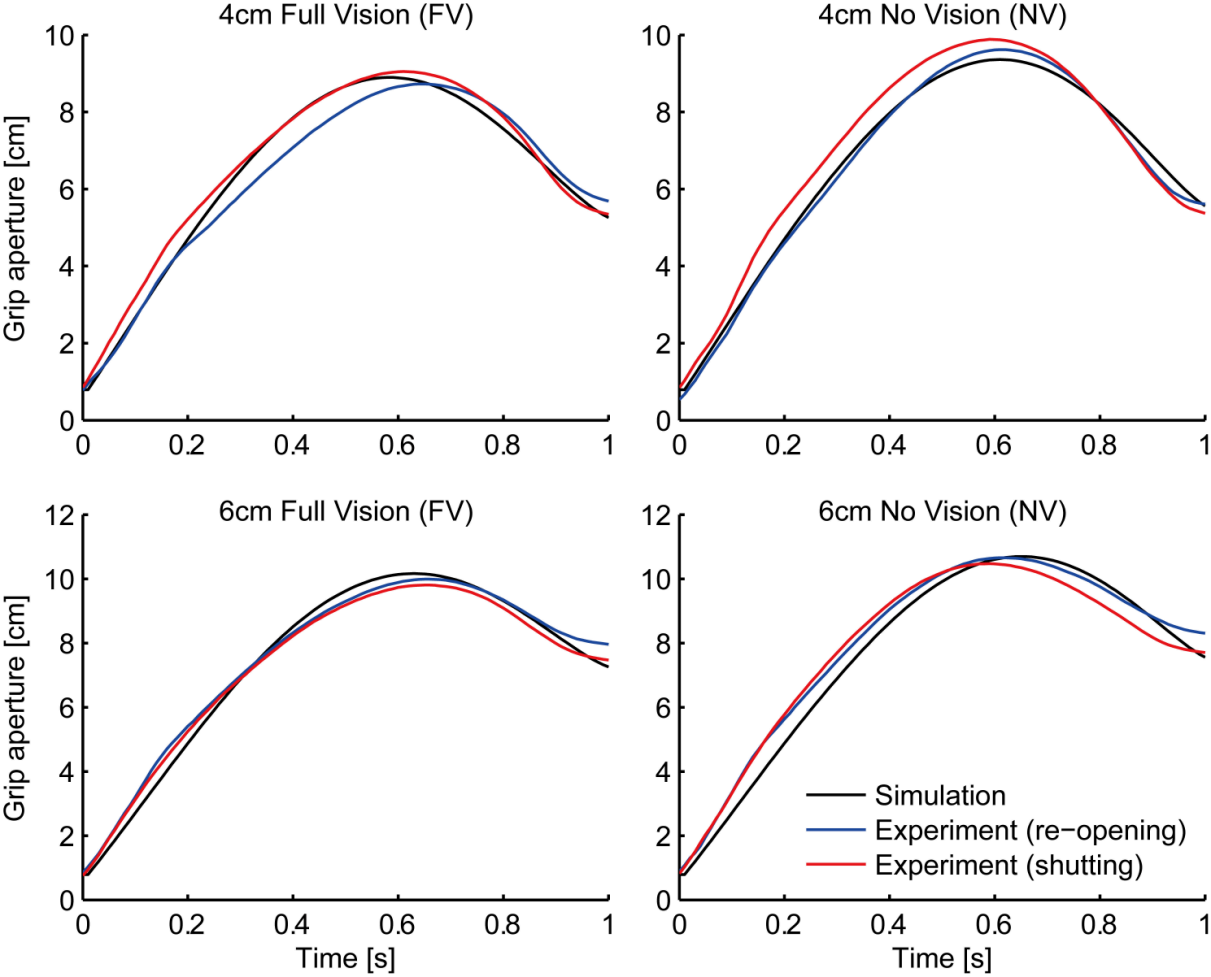
**Grip aperture for parameter fitting**. Profile of grip aperture from the experiment (Fukui and Inui, 2006; re-opening paradigm: blue lines; shutting paradigm: red lines) and the simulation (black) for 4 cm (top panels) and 6 cm (bottom panels) target objects in Full Vision (FV; left panels) and No Vision (NV; right panels) conditions.

As a result of fitting, parameter α was converged to zero. This means that we do not have to assume the relationship between the transport and aperture component in the variability domain. In other words, the aperture component considers the graspability of the target object only with respect to the variability (uncertainty) of the grip aperture and the object size (i.e., σ_*a*_ and σ_*o*_). This result is in line with the visuomotor channel hypothesis (Jeannerod, 1981, 1984, discussed below). The grip aperture profiles of the experiment and the simulation in the FV and NV conditions after parameter fitting are shown in Figure 4. The whole aperture profile is well-fitted to the experimental data.

The temporal development of the variables in the simulation of grasping toward a 4 cm target object in FV and 150S conditions is shown in Figure 3. As the hand approached the target object (Figure 3A), the predicted SD of the grip aperture decreased as the movement continued (Figure 3B) because the time for future prediction (i.e., the time left until the movement ends) decreased. Consequently, the target aperture decreased (Figure 3C) because the uncertainty of the final posture and the “safety margin” decreased. The profile of the grip aperture (Figure 3D) was generated by smooth following of the target aperture, exhibiting a peak at around 600 ms after movement onset. After the vision was occluded (Figure 3; the gray vertical line denotes 150-ms shutting), the visual noise increased (Figure 3E, dashed line) compared to the values obtained in FV conditions after sensory delay (i.e., 100 ms), and the predicted uncertainty of the final grip aperture (Figure 3B, dashed line), the target aperture (Figure 3C, dashed line) and the grip aperture (Figure 3D, dashed line) increased compared to the values obtained in FV condition.

**Figure 5:**
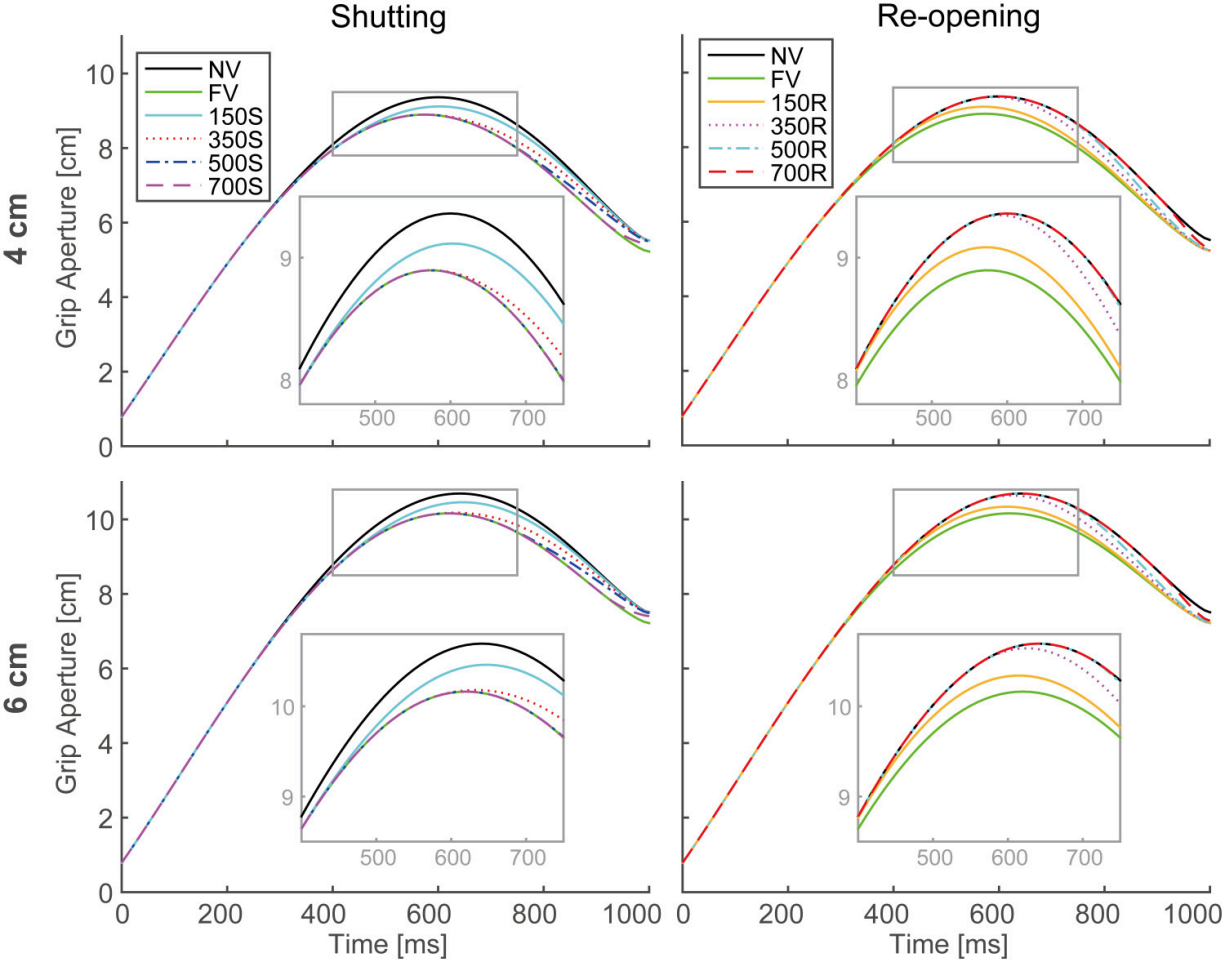
**Grip aperture profiles for simulation of visual occlusion**. **Upper left**: target object size is 4 cm in the shutting paradigm (SP), **Upper right**: 4 cm in the re-opening paradigm (RP), **Lower left**: 6 cm in SP, and **Lower right**: 4 cm in RP. In FV conditions, vision was constantly available during the movement, while in NV conditions, vision was constantly unavailable. In 150S, 350S, 500S, and 700S conditions, visual occlusion started 150, 350, 500, and 700 ms after movement onset, respectively. In 150R, 350R, 500R, and 700R conditions, vision was first unavailable and then recovered 150, 350, 500, and 700 ms after movement onset, respectively. For each panel, aperture profiles within gray rounded rectangles are magnified for precise view.

The output aperture profiles of the model's simulation in all visual conditions, with the parameters described in Table 1, are shown in Figure 5. For all conditions in both paradigms, the peak grip aperture appeared around 550–650 ms, as it did in the experimental results. Despite the lack of a mechanism for closing fingers upon touching the object, the model first opened the fingers larger than the object size and then closed them. This is because the predicted variability at the final phase of the movement decreased as the movement progressed. This decrease in the predicted variability arose primarily from the decrease in the duration to the end of the movement. Since, the Kalman filter calculates future aperture variability by repeatedly applying the incremental forward model (Appendix B), the later prediction points (i.e., prediction in earlier phase of the movement) will be more variable.

**Figure 6:**
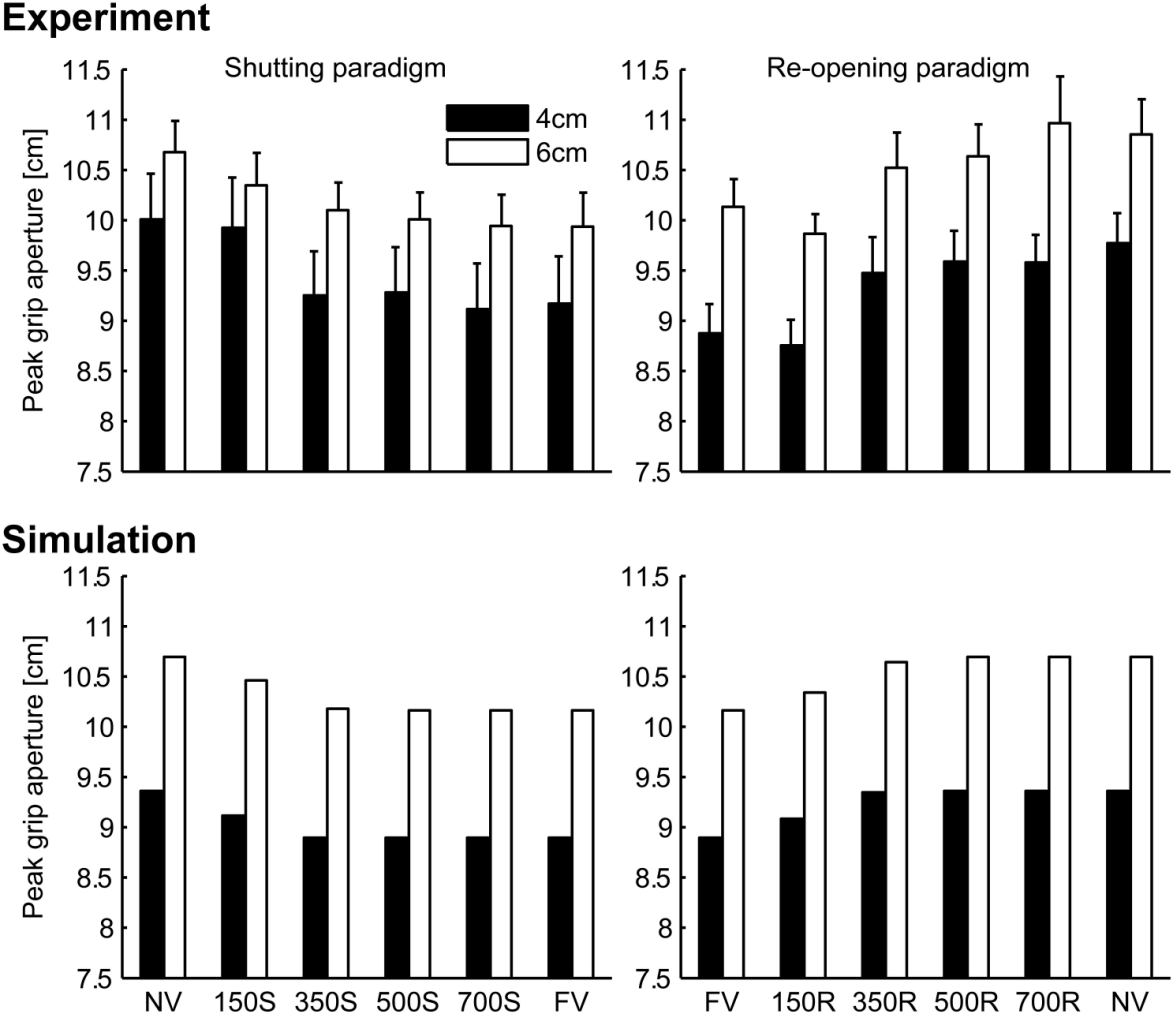
**Peak grip aperture in the simulation and the experiment**. **Upper left**: experimental result in shutting paradigm, **Upper right**: experimental result re-opening paradigm, **Lower left**: simulation result in shutting paradigm, and **Lower right**: simulation result in re-opening paradigm. Experimental data is from Fukui and Inui (2006). Error bars indicate the standard errors of the values between participants.

Peak grip apertures in both the simulation and the experiment are shown in Figure 6. In the simulation, the peak grip apertures in 350S, 500S, 700S, and FV (shutting paradigm) conditions were remarkably similar value, as well as in 350R, 500R, 700R, NV (re-opening paradigm). The peak grip apertures observed in the experiment in these conditions did not show statistical difference, indicating that visual availability after 350 ms does not affect the peak grip aperture. The simulation replicated the experimental result that showed that vision in the early phase of the movement is important for grasping. Note that the parameters in the model were determined by fitting to the aperture profile of the NV and FV conditions. Other conditions were not considered in the fitting. Therefore, no temporal information about the change in visual availability was contained in the data used for fitting. Even then, the simulation still reproduced the effect of early phase visual availability on the PGA. This occurred because the predicted variability mostly decreased in 350 ms in the model, and consequently, the visual conditions became insensitive to the aperture control.

**Figure 7:**
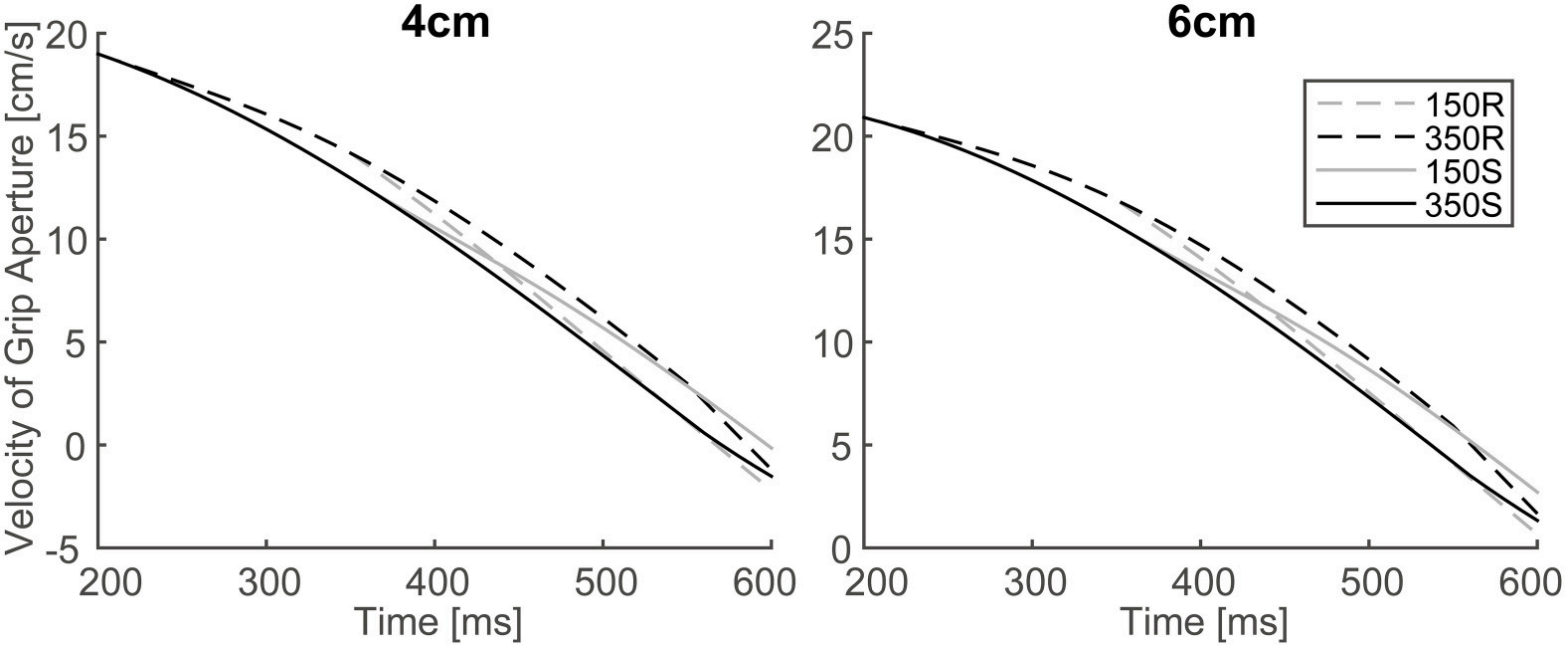
**Simulated velocity profile of grip aperture for conditions of 150S, 350S, 150R, and 350R**. Divergence between 150S (150R) and 350S (350R) appeared at approximately 400–450 ms after movement onset, as found in the experiment.

In order to verify the effect of online vision on the aperture profile, we investigated whether the simulated aperture velocity profiles between 150S (150R) and 350S (350R) conditions diverge as shown in the experiment of Fukui and Inui (2006). In the experiment, the difference between the aperture velocities for the 150S (150R) and 350S (350R) conditions was statistically significant after 400–475 ms following movement onset. The aperture velocity profile of the model's output in 150S, 150R, 350S, and 350R conditions for 4 and 6 cm target objects is shown in Figure 7. The time points when the aperture velocities diverged between 150S (150R) and 350S (350R) conditions were around 400–450 ms. Specifically, the motor response to the onset (recover) of visual occlusion at 150 ms in 150S (150R) conditions was expected to start at 350 (i.e., 150 + 200) ms after movement onset. Considering that the response becomes noticeable only after the divergence is large enough, the result that the divergence time is similar in the simulation and experiment (i.e., 400–450 ms) indicates that the sensorimotor delay used in the simulation (=200 ms) is reasonable in this prehension task.

**Figure 8:**
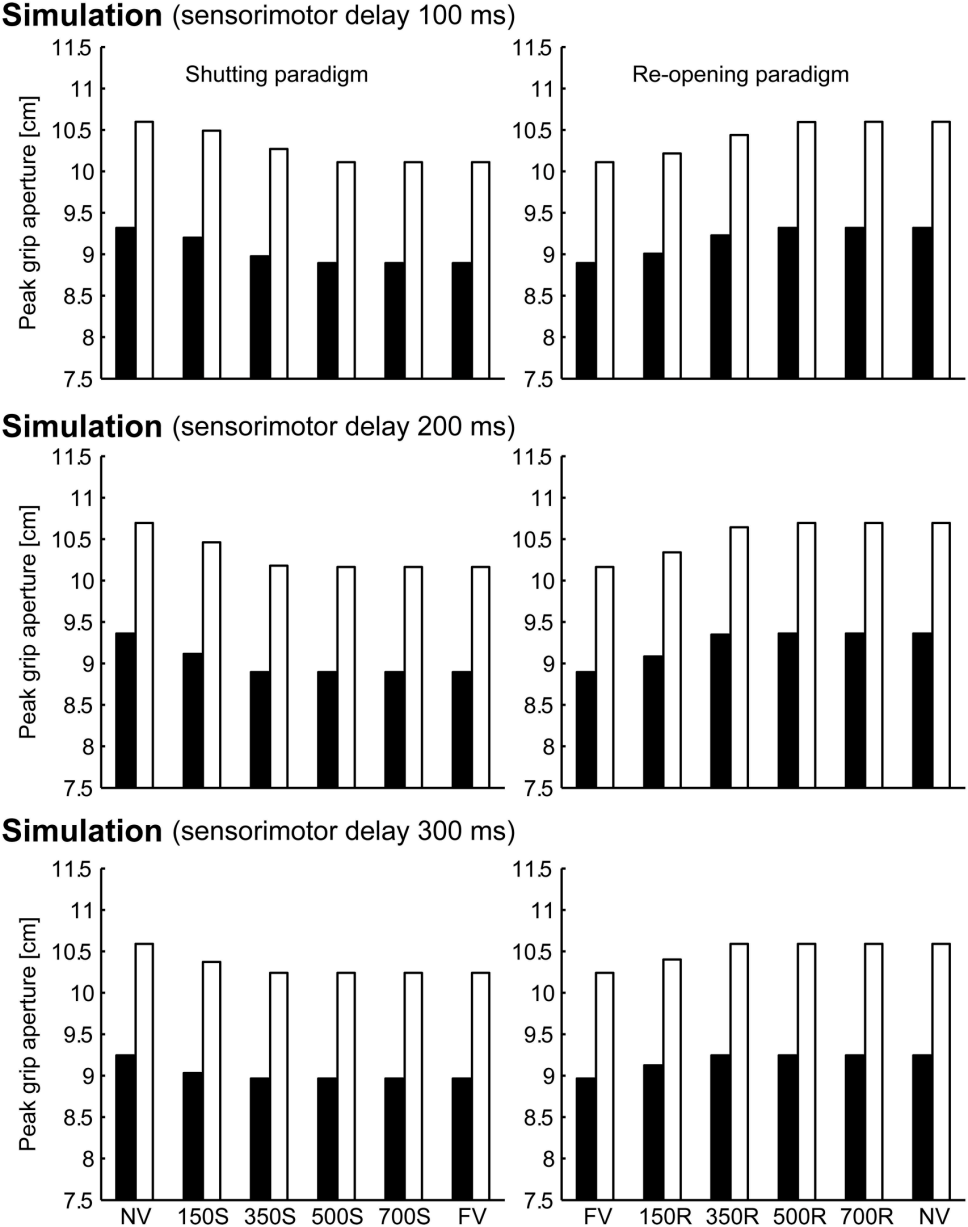
**Peak grip aperture in simulations with various sensorimotor delays**. **Top**: 100 ms sensorimotor delay. **Middle**: 200 ms sensorimotor delay. **Bottom**: 300 ms sensorimotor delay. **Left**: shutting paradigm. **Right**: re-opening paradigm. Note that the figures in the middle panels are identical to those shown in Figure 6.

**Figure 9:**
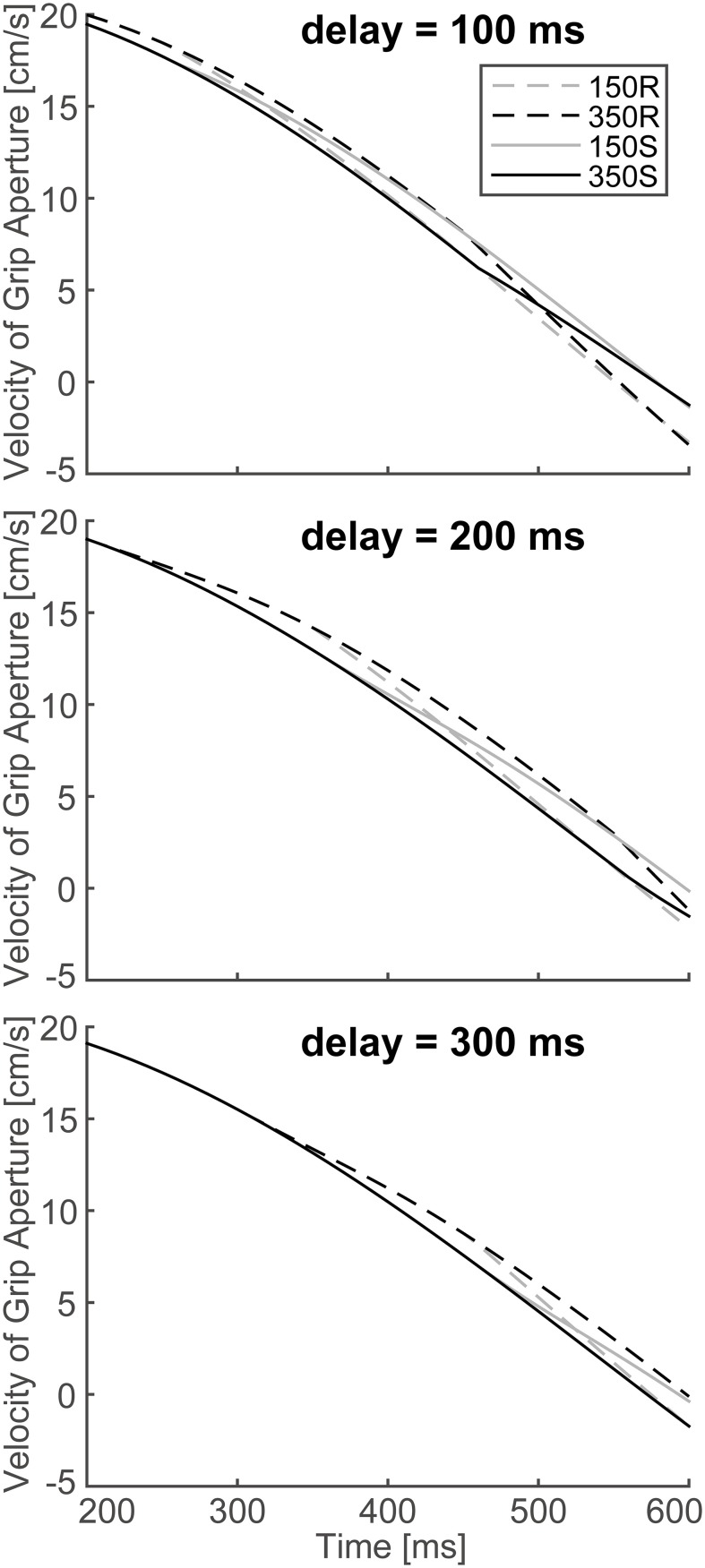
**Simulated velocity profile of the grip aperture for conditions of 150S, 350S, 150R and 350R, with 100, 200 and 300 ms sensorimotor delays**. The target object is 4 cm. **Top**: 100 ms sensorimotor delay. **Middle**: 200 ms sensorimotor delay. **Bottom**: 300 ms sensorimotor delay. Note that the figure in the middle panel is identical to that shown in Figure 7.

To verify the robustness of the model to the variability of sensorimotor delay, we performed the simulations with different sensorimotor delays (100 and 300 ms). We found similar effects of (non-)available duration of online vision on the PGA. Specifically, the PGA in 150S and 350S conditions increased with less sensorimotor delay (100 ms), and the PGA in 150R and 350R conditions increased with more sensorimotor delay (300 ms; Figure 8). The time of divergence in aperture velocity was dependent on sensorimotor delay (Figure 9). Among these three conditions, the simulation in the 200-ms delay condition was best fitted to the experimental result in terms of temporal divergence, and this 200-ms delay would be consistent with neural response latency suggested by Huettel et al. (2014).

These additional simulations indicated that this emerging effect of online vision on the PGA was not due to sensorimotor delay but to the control mechanisms of grip aperture with the predicted variability assumed in the present model.

**Figure 10:**
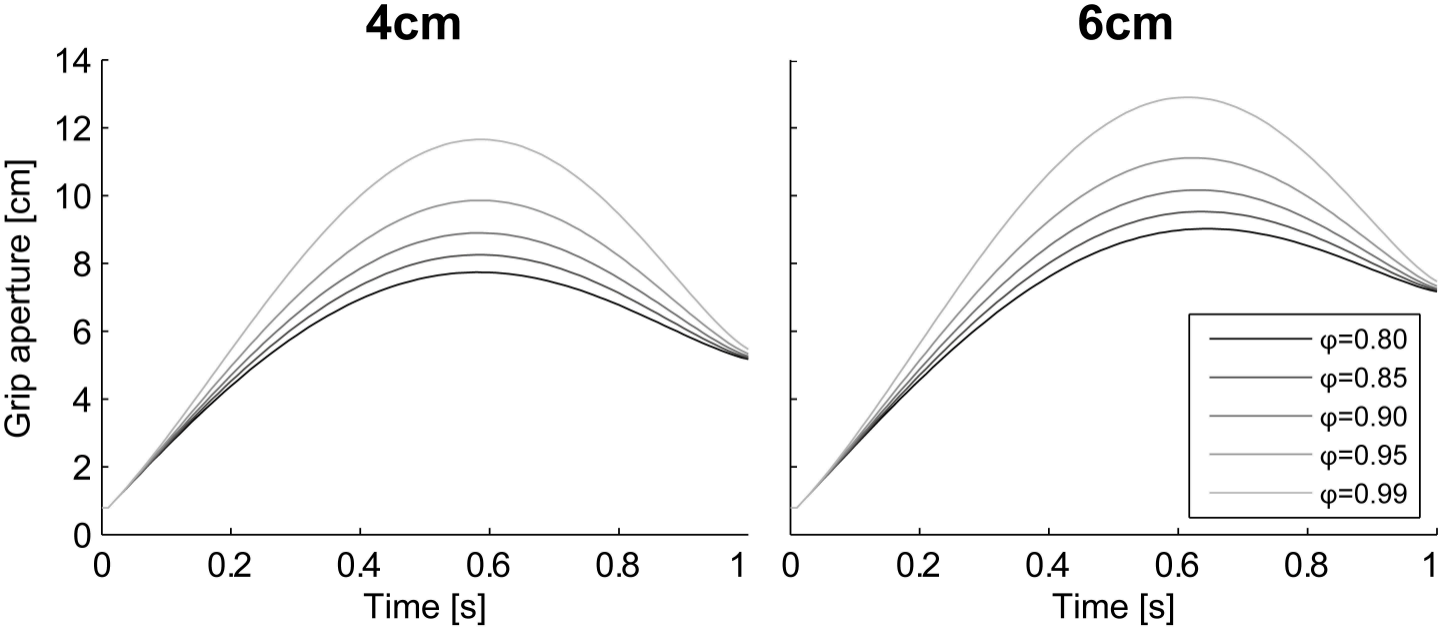
**Aperture profiles for various grasping probability ϕ**. Simulation result with ϕ = [0.8, 0.85, 0.9, 0.95, 0.99]. **Left panel**, 4 cm; **Right panel**, 6 cm.

The grasping probability parameter ϕ had a strong effect on the peak grip aperture. According to the simulation results in which various values were set to ϕ (= [0.8, 0.85, 0.9, 0.95, 0.99]) without changing other parameters, the grip aperture was larger when the probability requirement was larger (Figure 10). This is consistent with the intuition that a larger safety margin is preserved when humans grasp things more carefully.

Furthermore, we performed the simulations with minimum jerk instead of minimum acceleration because the minimum jerk was generally used as a criterion of movement smoothness in previous works. We found a slower decrease of the grip aperture in the final phase of the movement and an earlier time to PGA than observed in the experiment.

## Discussion

### Sensory and motor variability in models for reach-to-grasp movement

We proposed a model for reach-to-grasp movement that controlled grip aperture to compensate for predicted sensory uncertainty and motor variability. This is the first model that includes a control principle to explain how the grip aperture is arranged to grasp an object based on sensory uncertainty and motor variability. The formation of grip aperture was modeled in several previous studies. Hoff and Arbib (1993) divided the aperture formation into preshaping and finger closure phases. In the preshaping phase, aperture is opened toward the peak grip aperture that scaled with object size. They provide no explanation for how the peak grip aperture is actually determined. Smeets and Brenner (1999) modeled grasping as pointing of two fingers to the opposite side of the object, with the trajectory formed by minimum jerk principle. The overshoot of the grip aperture to the object size was generated by the constraint that the fingers had to approach the object orthogonally to the surface of the object. No explanation was given for determination of this “approaching parameter.” Ulloa and Bullock (2003) proposed a dynamic system model that included an equation to describe the influence of the transportation component on the aperture component. These models did not capture the function of sensory and motor variability in reach-to-grasp movement.

The fitting parameter α in the present model resulted in zero. This means that the prediction of wrist position variability is not necessary to determine the grip aperture in reach-to-grasp movements. However, it does not mean that there is no connection between the transport and aperture components; instead, a relationship between them in the time domain is implemented in the present model. Specifically, the grip configuration progresses by predicting future state variability at the movement end, which was pre-determined following the experimental instruction of movement time (i.e., 1000 ms). The movement time in the experiment was defined by the duration from the time when the wrist was released from the table to the time when the wrist started to move the target object approximately 30 cm towards the body. In other words, the grip configuration was partially regulated by the movement time, which is defined by the transport component. This type of temporal relationship between the two components has been modeled previously (Hoff and Arbib, 1993). Despite the assumption that the connection of these components was taken into account in the variability domain in the present model (i.e., the function of parameter α), the parameter that represents this connection resulted in zero, suggesting the visuomotor channel hypothesis (Jeannerod, 1981, 1984), which assumes an independence of each component in visuomotor transformation. Although the visuomotor hypothesis does not incorporate the framework of variability-based motor control, the present model suggests that this independence of each component could be applicable to the level of variability-based motor control. The assumption of the temporal coordination of these visuomotor channels (Hoff and Arbib, 1993) is also implemented in the present model.

One possibility for the result that α became zero after fitting could be the inappropriate assumption of the proportionality between the residual distance and the predicted variability of the hand position (Equation 15). However, the fact that the grip aperture profile was successfully reproduced without assuming the predicted variability of the hand position indicates that the variability-based model of reach-to-grasp movement without assuming a transport component has the capacity to parsimoniously explain the effect of online vision on the PGA shown by Fukui and Inui (2006). Nevertheless, incorporating a predicted variability of the hand position (which might not be describable by a simple function of the residual distance) into the model will contribute to a better understanding of reach-to-grasping movements. Further investigations are required to identify the relationship between the aperture and transport components in the variability domain.

As mentioned in the introduction, Simmons and Demiris (2006) have shown the importance of variability in the control of grip aperture. They modeled aperture control as a movement that goes through via-points for each finger. They found that when motor variability was dependent on the amplitude of a motor command, the via-points that resulted in the smallest variance at the end point of the movement were chosen. However, their model only focused on motor variability. To explain the effect of visual feedback in grasping, the function of sensory uncertainty also has to be modeled. Our model includes the Kalman filter for predicting future variability of the aperture, so that sensory uncertainty also plays a role in the control.

The present model suggests a new computational view expanding the conventional Bayesian sensorimotor framework: the online prediction of motor variability at the task end is used for feedback control. Previous studies have demonstrated that motor variability and sensory uncertainty are integrated on the basis of the Bayesian framework (or the Kalman filter) in order to enhance the task performance in the presence of sensory and motor noise during motor planning (Harris and Wolpert, 1998; van Beers et al., 2004; Trommershäuser et al., 2005), sensorimotor learning (Körding and Wolpert, 2004) and online control (Izawa and Shadmehr, 2008). In the present model, the target aperture is determined using the predicted variability of grip aperture at the movement end. This means that the online feedback controller changes the “immediate goal (target)” necessary for achieving the task depending on both the Bayesian estimation of the current state of the body and the environment and the Bayesian prediction of a future state. This feature of the present model explains how the effect of online vision during prehension varies according to the phase of the movement. In the early phase of the movement (0–350 ms), where the prediction of a far future (> 650 ms) is required, the predicted task end state is uncertain and the sensory feedback is important to reduce this uncertainty. We consider that this online variability prediction plays an important role in performing a grasping movement.

In the present model, motor noise is assumed to be a constant during the movement rather than signal-dependent noise (Harris and Wolpert, 1998). The controller used the predicted variability to determine a target aperture (a^*^), and it was impossible to predict the future variability of the grip aperture with signal-dependent noise, because the noise affects the target aperture, the motor commands used to achieve this target aperture, and, consequently, the control noise itself. A cost function is necessary to fix the motor command in the presence of signal-dependent noise. The parameter ϕ, which was set to 0.9 in the present study, might be determined with such a cost function and signal-dependent noise in future studies.

### Neural computation of variability

We calculated the future variability of the aperture and updated the variability due to sensory feedback by implementing the Kalman filter in our model. During the estimation of the visual image, forward and inverse calculations similar to the Kalman filter appear to be conducted during the visual process (Kawato et al., 1993). In a reaching movement, the states of the body and environment are estimated in a manner similar to the Kalman filter, in off-line adaptation (Izawa et al., 2008) as well as in online processing (Izawa and Shadmehr, 2008). The Bayesian inference necessary for the Kalman filter can be computed by the neural population that fires in a probabilistic manner (Ma et al., 2006), and a neural implementation of the Kalman filter has been suggested (Denève et al., 2007). However, the way that the Kalman gain is computed still remains to be resolved.

The neural substrates related to the calculation necessary for the Kalman filter have been speculated (Ogawa et al., 2007). During performance of a tracing or tracking movement using a computer mouse with an artificial delay introduced occasionally, the right posterior parietal cortex was activated in relation to the function that detects the error between the target and the mouse cursor. The right temporo-parietal junction was involved in state estimation of self-movement during visually guided movement. Unfortunately, these studies on neural computation related to the Kalman filter only investigated reaching movements, not grasping. However, motor control of the body is clearly related to the sensory and motor variability, and a reach-to-grasp movement also has to be explained in this manner. The present study demonstrated that a computational model based on mechanisms of variability prediction with the Kalman filter explains the online effect of vision during reach-to-grasp movement.

## Conclusions

We have proposed a computational model for a reach-to-grasp movement, where the state of the hand is estimated and predicted by Kalman filters, and a motor command is generated that establishes a target grip aperture that is sufficiently large, in the stochastic manner, in relation to the target object size. The simulations of the model reproduced the effect of visual occlusion on the PGA during grasping. Online control of the movement would therefore require: (i) internal prediction of future states of the body and its variability, and (ii) motor commands based on the prediction and task constraints. The model was constructed within the framework of optimal feedback control in the sense of predictive stochastic control under sensory and motor variability, although the control has not yet been fully optimized.

### Conflict of interest statement

The authors declare that the research was conducted in the absence of any commercial or financial relationships that could be construed as a potential conflict of interest.

## Appendix A

### Trajectory in minimum acceleration principle

Considering the trajectory from a current state *x*_*t*_ at current time *t* to a target state *x*_*T*_ at movement end time *T*, the cost to minimize is described by the following:

(A1) $$\begin{array}{l} C=\frac{1}{2}\int_{t}^{T}{\left(\frac{d^{2}x_{t^{\prime }}}{d{t^{\prime }}^{2}}\right)}^{2}dt^{\prime } \end{array}$$

In the similar manner as discussed by Flash and Hogan (1985) for the minimum jerk principle, the solution is given by a third order polynomial:

(A2) $$\begin{array}{l} x_{\tau }=a_{0}+a_{1}\left(\tau -t\right)+a_{2}{\left(\tau -t\right)}^{2}+a_{3}{\left(\tau -t\right)}^{3} \end{array}$$

where τ denotes the arbitral time during movement (*t* ≤ τ ≤ *T*).

The solution of (A2) can be obtained by applying boundary conditions, which specify the position *x*_*t*_ and the velocity ẋ_*t*_ at time *t*:

(A3) $$\begin{array}{l} a_{0}=x_{t}\;a_{1}={ẋ}_{t}\\ a_{2}=\frac{3\left(x_{T}-x_{t}\right)}{{\left(T-t\right)}^{2}}-\frac{{ẋ}_{T}+2{ẋ}_{t}}{\left(T-t\right)}\\ a_{3}=-\frac{2\left(x_{T}-x_{t}\right)}{{\left(T-t\right)}^{3}}-\frac{{ẋ}_{T}+{ẋ}_{t}}{{\left(T-t\right)}^{2}} \end{array}$$

Taking the derivative of (A2),

(A4) $$\begin{array}{l} x_{\tau }=a_{1}+{2a}_{2}\left(\tau -t\right)+{3a}_{3}{\left(\tau -t\right)}^{2} \end{array}$$

The velocity profile in the minimum acceleration principle, Equations (4) and (5), is obtained from (A3) and (A4).

## Appendix B

### The kalman filter with time delay

The system dynamics are described by Equations (11) and (12). The estimation of the state at time *t*_1_ based on the observation until time *t*_2_ is denoted by $x_{t_{1}|t_{2}}$, and the covariance of the estimation error is denoted by $P_{t_{1}|t_{2}}$. Because of sensory delay, the observation at time *t* is used to correct the estimation of state at time *t* − *d*_*s*_:

(B1) $$\begin{array}{l} K=P_{t-d_{s}|t-1}H^{T}{\left[HP_{t-d_{s}|t-1}H^{T}+R_{t}\right]}^{-1} \end{array}$$

(B2) $$\begin{array}{l} {\hat{x}}_{t-d_{s}|t}={\hat{x}}_{t-d_{s}|t-1}+K\left(y_{t}-H{\hat{x}}_{t-d_{s}|t-1}\right) \end{array}$$

(B3) $$\begin{array}{l} P_{t-d_{s}|t}=P_{t-d_{s}|t-1}-KHP_{t-d_{s}|t-1} \end{array}$$

Here, [□]^*T*^ denotes a transposition of the vector or matrix, while [□]^−1^ denotes an inverse matrix. The estimation of the state at *t* is calculated by corrected estimation ${\hat{x}}_{t-d_{s}|t}$ and a motor command that has already been output, by applying the following in order of *i* = −*d*_*s*_, …, −1:

(B4) $$\begin{array}{l} {\hat{x}}_{t+i+1|t}=F{\hat{x}}_{t+i|t}+Du_{t+i-d_{m}} \end{array}$$

(B5) $$\begin{array}{l} P_{t+i+1|t}=FP_{t+i|t}F^{T}+GQ_{t+i-d_{m}}G^{T} \end{array}$$

The motor command generated at time *t* − 1 arrives at the end effector at time *t* + *d*_*m*_ − 1 because of the motor delay. Therefore, the state estimation ${\hat{x}}_{t+d_{m}|t}$ at time *t* + *d*_*m*_ can be calculated at time *t* by applying (B4) and (B5) for *i* = 0, …, *d*_*m*_ − 1. As mentioned in Section Formulation of Aperture Size Control, this estimation is necessary for generation of the motor command *u*_*t*_. Equation (B5) is also used to predict future variability at the end of movement. As variability is predicted farther into the future, the predicted variability increases because repetitive application of Equation (B5) accumulates the motor variability *Q*_*t*_.
